# Microglial iron trafficking: new player in brain injury

**DOI:** 10.55730/1300-0144.5481

**Published:** 2022-08-06

**Authors:** Elif KELEŞ, Hasan Hüseyin KAZAN, Arzu ARAL, Hayrunnisa BOLAY

**Affiliations:** 1 Neuropsychiatry of Education, Research and Application Center, Faculty of Medicine, Gazi University, Ankara, Turkey; 2 Department of Radiology, Feinberg School of Medicine, Northwestern University, Chicago, USA; 3 Department of Medical Genetics, Faculty of Medicine, Gazi University, Ankara, Turkey; 4 Neuroscience and Neurotechnology Center of Excellence (NÖROM), Ankara, Turkey; 5 Department of Immunology, Faculty of Medicine, İzmir Demokrasi University, İzmir, Turkey

**Keywords:** Neonatal brain development, brain injury in neonates, microglia, iron metabolism, lipid peroxidation, ferroptosis

## Abstract

Neonatal brain injury is a significant reason of neurodevelopmental abnormalities and long-term neurological impairments. Hypoxic-ischemic encephalopathy and preterm brain injury, including intraventricular hemorrhage are the most common grounds of brain injury for full-term and preterm neonates. The prevalence of hypoxic ischemic encephalopathy varies globally, ranging from 1 to 3.5/1000 live births in high-resource countries and 26/1000 in low-resource countries. Preterm birth’s global incidence is 15 million, a significant reason for infant mortality and morbidity, permanent neurologic problems, and the associated social and economic burden. The widespread neurodevelopmental effects of neonatal brain injury could have an unfavorable impact on a variety of aspects of cognitive, linguistic, behavioral, sensory, and motor functions. Brain injury occurs via various mechanisms, including energy deprivation, excitatory amino acids, mitochondrial dysfunction, reactive oxygen species, and inflammation giving rise to different forms of cell death. The contribution of microglial activity in neonatal brain injury has widely been underlined by focusing on cell death mechanisms since the neuronal death pathways during their development are distinct from those in the adult brain. Iron accumulation and lipid peroxidation cause a relatively novel type of regulated cell death called ferroptosis. Neonates generally have biochemical iron inequalities, and their antioxidant potential is highly restricted, implying that ferroptosis may be significant in pathologic conditions. Moreover, inhaled nitric oxide therapy in infants may lead to microglial inflammation via ferroptosis and neuronal injury in the developing brain. This review article aims to summarize the studies that investigated the association between neonatal brain injury and iron metabolism, with a particular emphasis on the microglial activity and its application to the inhibition of neonatal brain injury.

## 1. Introduction

Neonatal brain injury is a significant reason for children’s developmental problems and persistent neurological deficits. Hypoxia and ischemia are central to the development of brain injury that occurs in full-term and preterm neonates [1, 2]. Hypoxic-ischemic encephalopathy (HIE) is a common problem with a global prevalence ranging from 1 to 3.5/1000 live births in high-income countries and 26/1000 in low-resource countries [3]. Preterm birth’s global incidence is 15 million and it is a significant reason for infant mortality and morbidity with permanent neurologic problems and the associated social and economic burden [4]. HIE is also associated with other comorbid neurological disorders such as seizures and learning, behavioral, visual, cognitive, and motor disorders [5].

Alarmingly, the incidence of HIE in preterm infants is higher (4–48 per 1000 preterm neonates), representing that it accomplishes a major task in the pathogenesis of preterm brain injury [6, 7]. Currently, there are no approved therapies for preterm brain injury, and hypothermia treatment (HT) is the only approved therapy for term infants in terms of HIE [4, 8]. Still, current HT practice has decreased HIE-related mortality from 25% to 9% and cerebral palsy (CP) from 20% to 16% [9]. Studies have revealed that HT did not help all children and that certain neurodevelopmental problems remain even in the absence of CP. Thus, further therapies are needed to improve long-term outcomes [10, 11].

The development of novel therapies for the treatment of neonatal brain injury strictly requires the identification of novel cellular pathways, particularly cell death mechanisms [12]. Reactive oxygen species (ROS), inflammation, excitatory amino acids, and mitochondrial dysfunction are among some of these cellular mechanisms that lead to brain injury via cell death [11, 13–15]. The neonatal brain has an increased oxygen consumption, polyunsaturated fatty acid (PUFA) concentrations, and an insufficient antioxidant system, making it particularly defenseless to oxidative damage [16, 17]. Hence, it is fundamental to focus on the oxidation-based molecular pathways to understand and deal with brain injury in neonates.

**Figure 1:**
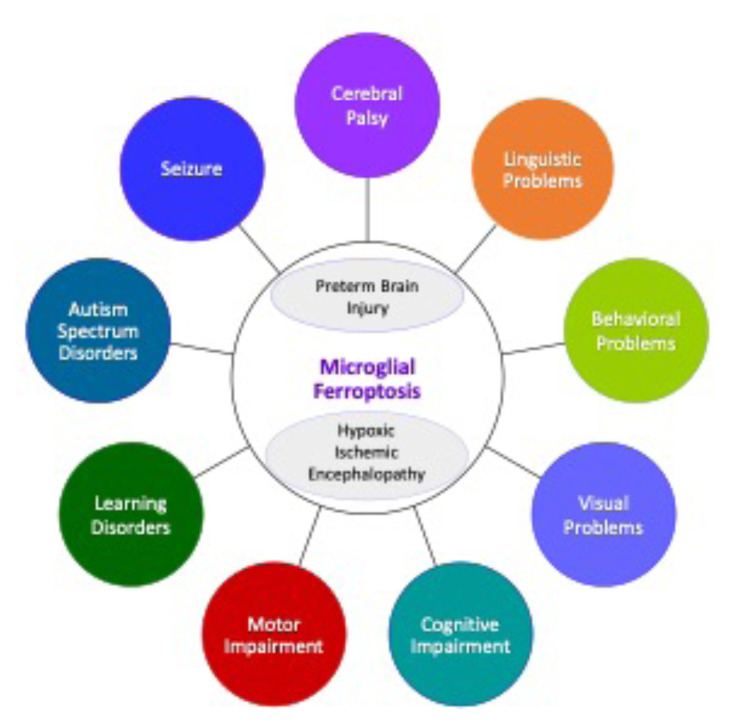
Flow diagram outlining the clinical outcomes of microglial ferroptosis resulting from preterm brain injury and hypoxic ischemic encephalopathy.

Microglia are the immune cells that act in central nervous system (CNS) inflammation. They account for around 10%–15% of all brain cells. Although these cells are incredibly well-regulated, any abnormalities in their functions may instigate neurodegeneration and perinatal and neonatal brain injuries, particularly by cell death mechanisms in which pyroptosis is dominant [18–20]. However, investigating other death mechanisms in microglia is critically important to fully explore the etiology of microglia-mediated brain injuries. Neonatal brain injury leads to different long-term clinical outcomes, including CP, motor and intellectual impairment, behavioral and visual problems, learning disorders, seizure, and autism spectrum disorders [5, 21–24] (Figure 1). This review aims to assess the literature on neuronal iron metabolism, lipid peroxidation, and ferroptotic mechanisms, mainly in microglia that contribute to neonatal brain injuries.

### 1.1. Iron metabolism, ferroptosis, and neuronal cell death

As a vital mineral, iron is involved in many metabolic activities, including electron and oxygen transport, storage, mitochondrial function, and cellular development. These biochemical enzymatic reactions are executed by iron-containing proteins, including hemoglobin and heme-containing and iron-sulfur enzymes. The imbalance in the iron metabolism could result in local or systemic drawbacks via a specific cell death mechanism called ferroptosis [25, 26].

Ferroptosis is a regulated cell death due to iron accumulation and lipid peroxidation [25–27]. Despite its name, ferroptosis is not a kind of cell suicide but rather a form of cell sabotage that occurs throughout normal cell activities to adapt to stimulus and physiological changes [25, 26]. As a result of its dysregulation or instability, ferroptosis is frequently involved in a variety of physiological and pathological processes, including neurotoxicity, cancer, neurodegenerative disorders and reperfusion injury [26, 27]. Ferroptosis is fundamentally different from other types of cell death in terms of evolution, biochemistry, genetics and morphology [25, 26, 28, 29].

At the molecular level, the initial process of ferroptotic death is mediated by hydroxyl radicals (OH), which are generated during the Fenton reaction, in which Fe^2+^ iron is oxidized to Fe^3+,^ resulting in a highly reactive pro-oxidant OH radical. The OH radical reacts with PUFAs to produce lipid radicals. The lipid radical reacts with molecular oxygen to constitute lipid peroxyl radicals, which spread the reaction by damaging surrounding lipid molecules, producing lipid hydroperoxides and more lipid radicals. These reactions continue until the antioxidant enzyme glutathione peroxidase 4 (GPX4) catalyzes the reaction by donating hydrogen atoms generated during glutathione (GSH) synthesis to produce nonradical lipid alcohols under normal conditions. The enzymatically triggered process of lipid peroxidation initiates ferroptosis, which requires phosphatidylethanolamine (PE)-containing arachidonic acid (AA), one of the major phospholipids that undergo oxidation. In this process, Acyl-CoA synthetase long chain family member 4 (ACSL4) acetylates free PUFA (AA) with coenzyme A to produce AA-CoA, which is then integrated into the membrane phospholipid PE via the enzyme lysophosphatidylcholine acyltransferase 3 (LPCAT3). Lipoxygenases (LOXs) then oxidize PE-AA to form PE-AA-OOH, which promotes ferroptosis [26, 30–34]. As revealed by studies aimed at potential mechanisms of different ferroptosis enhancers, system X, as well as GPX4 inhibition, could all lead to a decline in GSH levels and the consecutive release of ROS, particularly as a result of increased lipid peroxidation levels and ferroptosis [25, 26, 29].

Prior to identifying ferroptosis, deferoxamine (DFO) was crucial in preventing brain oxidative stress and death. DFO was already proven to be efficient in the suppression of CNS cells from death before ferroptosis was discovered [35, 36]. However, the factor driving this function remains unknown. Recent studies on ferroptosis could give an insight into this involvement [30, 35, 37, 38].

Ferroptosis is involved in neuronal cell death generated by adult ischemia and intraventricular hemorrhage, and suppression of the ferroptosis protects the neuronal cell from death [30, 36, 39, 40]. Nevertheless, despite the fact that there have only been a few studies demonstrating ferroptosis regarding neonatal brain injury, the evidence implies that ferroptosis would more likely occur in the neonatal brain [41–43]. For instance, in a study where cultured oligodendrocytes were exposed to cysteine-free conditions to mimic periventricular leukomalacia in vitro, GSH was depleted, and cell death was increased, which was also reversed by ferrostatin [32, 44].

Numerous antiferroptotic agents have been discovered [29, 30, 44–47]. First-generation inhibitors of ferroptosis include ferrostatin-1 (Fer-1) and liproxstatin-1 (Lip-1; [44]. Lipid peroxidation is prevented by those inhibitors, which react with and donate hydrogen atoms to peroxyl radicals in lipids [31, 44, 46, 47]. Reduced neurological impairments, infarct volume, and neuronal death after a stroke have been shown to be suppressed by inhibition of ferroptosis by Fer-1 and/or Lip-1, which also reversed the changes in GPX activity and iron accumulation [30, 47, 48]. Additionally, oligodendrocyte cells have been protected from cuprizone-induced demyelination by Fer-1 [41]. These inhibitors are lipid radical scavengers currently being studied for their role in neurodegenerative pathology [30, 48, 49]. In parallel, preventing neonatal brain injury could be one of their applications [26, 27, 30, 39, 42, 44, 48, 50–53].

### 1.2. Microglia-mediated brain injury, iron metabolism, and ferroptosis

Neonatal brain injury is presented by a unique encephalopathy that arises in the early days of life and a major cause of life-long neurological impairment [54]. There would be numerous molecular reasons for neonatal brain injury, which are reviewed and emphasized the involvement of cell death mechanisms [3, 18–20].

Molecular mechanisms behind the association between microglia, iron metabolism/ferroptosis, and brain injury, limitedly regarding the developmental stage, have recently been focused on the literature [55]. The studies underlined the pro-inflammatory role of ferroptosis in the microglia [40, 56–61]. However, we regard the contribution of iron metabolism and ferroptosis to brain injury should be evaluated in terms of the developmental stage since the brain development in adults differs from that of the neonates [16, 30, 62].

In the CNS, macrophages and microglia are crucial for cell function and immunological protection. CNS-resident microglia and peripheral macrophages mobilize to act on an injury or infection. This engagement reflects the most critical activities of CNS as well as their antiinflammatory or resolving functions. There is a wide range of features displayed by macrophages, microglia, and a well-defined multipattern involving inflammatory/classical (M1) and resolution/alternative (M2) macrophages/microglia activation [63].

The iron uptake patterns of M1 and M2 macrophages/microglia are conceptually distinct. Neonatal brain injury is hypothesized to arise due to an excess level of iron in the brain’s most sensitive areas during pregnancy. It is called neuroinflammation to describe the inflammatory reactions triggered by the brain’s innate immune system. Even though brain inflammation shares many characteristics with peripheral one, it is distinguished by CNS-specialized cells like neurons, microglia, astrocytes, endothelial cells and pericytes, which are not found in peripheral inflammation [15, 18, 19, 49]. In neuroinflammatory situations, the blood-brain barrier (BBB) is compromised, allowing peripheral inflammatory cells such as macrophages to move into the brain. In addition to their effects on cell differentiation, iron also influences cell homeostasis [18, 49].

Iron could transit the BBB via the transferrin-1 (TFR1), divalent transporter-1 (DMT-1), ferroportin-1 (FPN-1) route, a model similar to that used by cells in the periphery [64]. Iron is transmitted to the BBB by FPN, which releases Fe^2+^ from the cellular membrane of microvascular endothelial cells, promptly oxidized to Fe^3+^ by ceruloplasmin, released into the interstitium through the astrocyte, and afterwards trapped by transferrin. Fe^3+^ ions in the CNS are linked to small molecules that astrocytes distribute into the brain [30, 39, 64]. Contrary to the periphery, CNS encompasses significantly more nontransferrin-bound iron (NTBI). TFRs are overexpressed in neurons in their normal state and the majority of the iron comes from transferrin. Astrocytes produce DMT-1 and absorb Fe^2+^ ions, which are present in the form of NTBI. TFRs facilitate microglia to obtain transferrin-bound iron (TBI). Neurons and other glial cells are exposed to NTBI due to higher DMT-1 levels in an inflammatory setting. Some factors can upset this iron balance, resulting in an iron deficiency or excess. By the accumulation of iron in the CNS, iron regulatory protein 1 (IRP1) utilizes the Fe-S complex to gain aconitase activity, whereas IRP2 is destroyed. As a result, IRPs lose their affinity for iron responsive elements (IREs), leading to mRNA breakdown with Fe^3+^ ions [30, 39, 64].

The iron handling patterns of M1/M2 macrophages are radically different and surprising. Reduced IRP binding activity, a lower cytoplasmic labile iron pool (cLIP), lower levels of TFR1 and FPN1, and more significant amounts of ferritin heavy (Ferritin-H) found in M1 macrophages. M1 macrophages are less capable than M2 macrophages in terms of iron uptake and transfer. M1 macrophages respond to extracellular iron imbalance or overload with a limited capacity [51, 65, 66].

Neuroinflammation and iron accumulation, linked by a complicated network of molecular interactions, form a toxic circuit that propagates the neurodegenerative process. Iron stimulates the proinflammatory M1 phenotype in microglia and macrophages as defined by inducible nitric oxide synthase (iNOS) production, which is required for adaptive remodeling of iron homeostasis in M1 microglia/macrophages [63, 67]. These modifications possibly enable neuroinflammation to survive, albeit in an environment of increased oxidative stress, which could be life threatening to neurons. Additionally, NO can degrade the Fe-S complex in c-aconitase, assisting IRP1 while driving up mitochondrial iron reserves and oxidative stress in iron-deficient environments, culminating in neuronal death [30, 39, 64]. Importantly, accumulated iron, attributed to hypoxic conditions at the cellular level, particularly in microglia, stimulates ROS production, cytokines and oligodendrocyte apoptosis. Microglia prevents excessive iron accumulation in immature oligodendrocytes following hypoxic injury [68].

## 2. Discussions

In our previous study, primary mouse microglia and astrocytes in culture were exposed to 12 h of hypoxia with or without mild hypothermic preconditioning. Iron importer proteins, DMT-1 and TFR1, increased their mRNA expression in response to hypoxia, and hypothermic preconditioning maintained the elevation of DMT-1 in both glial cell types. Ferroportin expression increased as a glial cell survival factor after hypoxia. Hypothermic preconditioning promoted the development in both cell types, particularly in astrocytes. Following hypothermic preconditioning, interleukin-10 (Il-10) levels significantly increased in cells. We demonstrated that hypothermic preconditioning before hypoxia reduced neurotoxicity and inhibited the expression of the ferritin light chain, primarily by modifying transport protein expression. This may be beneficial by mitigating the iron-related detrimental effects following hypoxic injury. The fundamental mechanism that is responsible for therapeutic hypothermia is not yet fully understood. Hypoxia increased iron overload in the mixed glial cells and ferritin expression in microglia and astrocytes via iron transport. We infer that hypothermic preconditioning may provide a protective strategy during hypoxia through iron homeostasis. Further research into in vivo models in terms of the protective mechanism of therapeutic hypothermia in HIE may give a better understanding of this in the near future [69].

Following hypoxia-induced brain injury, the transcription complex of hypoxia-inducible factor 1 subunit alpha (HIF-1α) is activated, resulting in the activation of multiple genes that enable cells to adjust to hypoxia and, therefore, recover from cell death. HIF-1α regulates TFR1 and DMT1 expressions in neurons, astrocytes, and microglia [70–72].

In another study, we found that 24-h stimulation of glyceryl trinitrate (GTN), which serves as a NO donor and a trigger of iNOS generation, resulted in considerable NO production in primary mouse microglia, astrocyte, and meningeal cell cultures. In this work, GTN stimulation increased the iron absorption in microglia, which was previously reported. Ferritin light chain expression in microglia was promoted by GTN, and ferritin heavy chain expression was stimulated in astrocytes in our study. In addition to the iron that is taken up by transport proteins, microglia can obtain iron from phagocytosis from the environment. As our research has already demonstrated, microglia were more susceptible to iron deposition than astrocytes [73].

As a clinical vignette in neonatology, inhaled NO (iNO) is commonly used in both preterm and term infants in order to treat primary persistent pulmonary hypertension resulting from different clinical situations, including sepsis, hypoxic ischemic encephalopathy, meconium aspiration syndrome, respiratory distress syndrome, congenital diaphragmatic hernia, pulmonary hypoplasia and congenital heart disease [74]. Despite the lack of evidence of iNO’s efficacy or safety and consensus statements against its routine utilization in preterm infants, its use has increased [75]. There is an ongoing discussion on the neurodevelopmental effect of the iNO in infants and long-term neurodevelopment impairment [74–76]. Endogenous NO affects risk factors in the developing normal and injured brain [77]. The neuronal isoform NOS-1 induces either positive benefits through vascular adaptation or detrimental cellular consequences during ischemia [77, 78]. The influence of exogenous NO on the developing CNS is highly controversial under normal and stressful situations [77, 79]. Inhaled NO increases vascular recruitment and diminishes brain injury only during ischemia but not reperfusion [77]. We also speculate from our previous study and current scientific evidence that iNO therapy in infants may lead to microglial inflammation through the ferroptosis and contribute to neurodevelopmental impairment. In line with our hypothesis, NO inhibitors in the early reperfusion/reoxygenation phase could alleviate the neonatal brain injury due to hypoxia and ischemia [52]. These observations highlight the need for further preclinical and clinical research on the effects of inhaled NO in neonatal brain injury.

Discovering the molecular route that underlies the interaction between inflammatory processes and iron overload will boost the development of novel treatment methods for interrupting this loop in neonatal brain injury [39, 43]. Hepcidin therapy, which prevents iron from penetrating into the CNS, and classical brain iron chelation are feasible treatment options for these severe illnesses [30, 32, 48].

**Figure 2:**
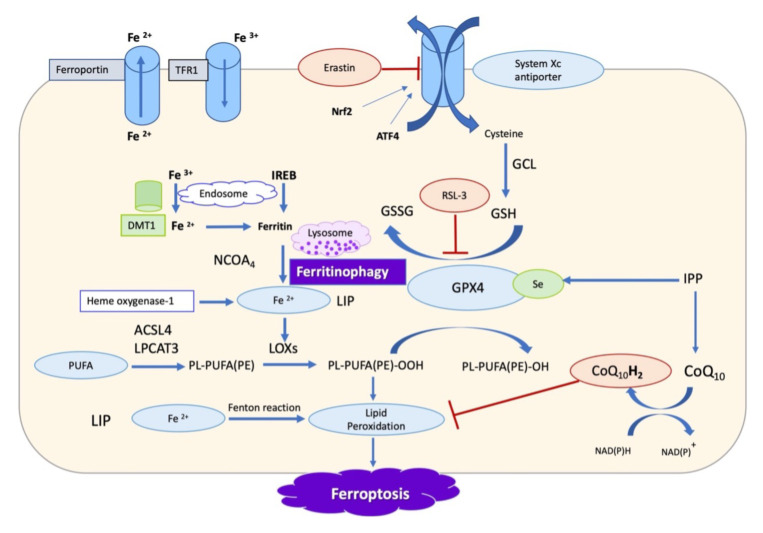
Components of ferroptosis pathway are shown graphically. Cells that are engaged in iron-induced free radical production (left), as well as GSH/GPX4 dysregulation (in the middle), have been found to be implicated in this process. To induce ferroptosis, erastin or RAS-selective lethal 3 (RSL3) inhibits the amino acid antiporter in system Xc and GPX4, resulting in lower activity of GPX4. The active site of GPX4 contains selenocysteine. Selenium-tRNA maturation requires the production of Isopentenyl Pyrophosphate (IPP). The plasma membrane’s ferroptosis suppressor protein 1 (FSP1) contains oxidoreductase activity, which lowers coenzyme Q levels and minimizes L-OOH accumulation. Lipoxygenase requires iron as a cofactor, which is provided by HO-1, ferritinophagy, and the labile iron pool (LIP). TFR1: transferrin-1, IREB: Iron-responsive element-binding protein-1, ACSL4, acyl-CoA synthetase long-chain family member 4; GPX, glutathione peroxidase; GSH, glutathione; LOXs, lipoxygenases; LPCAT3, lysophosphatidylcholine acyltransferase 3; PL, phospholipids; PL-AA-OOH, lipid hydroperoxides; NCOA4: nuclear receptor coactivator 4, PUFA : polyunsaturated fatty acid, Nrf-2: nuclear factor erythroid 2–related factor 2, ATF4: Activated transcription factors-4, Se: Selenium, CoQ_10_:Coenzyme Q_10_, CoQ_10_H_2 :_ Reduced coenzyme Q_10,_ NAD(P)H: nicotinamide adenine dinucleotide phosphate.

**Figure 3:**
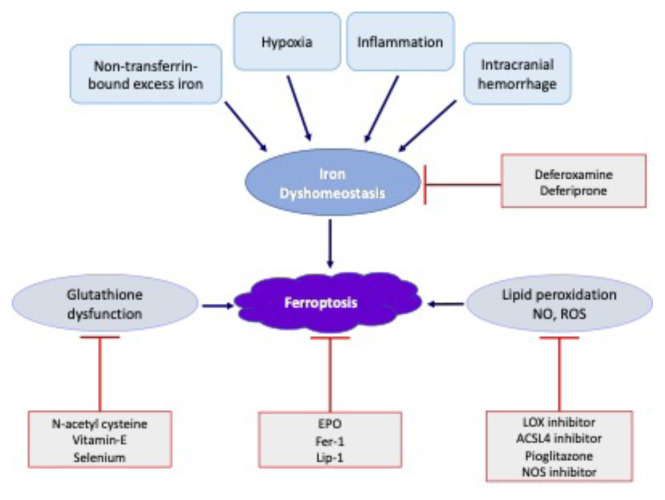
Flow diagram outlining the underlying mechanisms contributing to neonatal brain injury and potential prevention mechanisms. Graphical illustration of the main pathophysiological mechanisms underlying the development of brain injury in the neonates, namely hypoxia-ischemia-reperfusion, hypoxia-hyperoxia, inflammation, hemorrhagic insults, and PBIs. GSH depletion, glutathione S-transferase (GST) deformation, and reduced glutathione peroxidase (GPX) expression are observed in ferroptosis in neonatal brain injury. Iron accumulation potentiates inflammation, ROS, and NO associated with preterm and neonatal brain injury. Inflammation, excess iron (nontransferrin-bound iron), intracranial hemorrhage in preterm infants, and hypoxia in preterm and term infants induce cell death via oxidative stress and lipid peroxidation. Nitric oxide induces hypoxic ischemic injury in the neonatal brain via the disruption of neuronal iron metabolism. Different compounds may also show positive results in preterm brain injury and hypoxic ischemic encephalopathy, acting by attenuating different components of ferroptosis. EPO: Erythropoietin, FER-1: Ferrostatin-1, Lip-1: liproxstatin-1, LOX: Lipoxygenases, ACSL4: Acyl-CoA synthetase long chain family member 4, NOS inhibitor: nitric oxide synthase inhibitor.

It is known that preterm birth is related to an elevated risk of intracerebral hemorrhage (ICH). Reduced birth weight and gestational age are associated with an increased prevalence of ICH. ICH affects 15% of extremely preterm infants, with more than 50% of all preterm infants undergoing posthemorrhagic ventricular dilatation and approximately 30% acquiring long-term neurodevelopmental sequela [22, 24, 80]. Infants suffering from ICH are at risk of neurological impairment but no effective treatment is available. ICH-induced brain injury, which comprises primary physical damage produced by the hemorrhage and secondary damage caused by the action of extremely toxic substances released by the hemorrhage, is exacerbated by iron formation, resulting in ventricular dilatation. These components include free iron and cell-free hemoglobin, which significantly contribute to ventricular dilatation [81–83]. In addition, the use of iron-chelator treatment following an ICH is firmly established, proving the involvement of iron in the onset and progression of brain damage following an ICH [38]. Significant evidence has been consolidated indicating that fibrin components, including hemoglobin, fibrinogen, and iron, are important in developing secondary damage following an ICH [83–85]. In recent years, neonatologists have focused on hemoglobin and iron-induced neuronal toxicity, as hemoglobin and its metabolites are cytotoxic [86]. They have the potential to create oxidative stress and inflammation in the body [87]. Heme oxygenase (HO-1) degrades hemoglobin in the brain and releases iron, carbon monoxide and biliverdin into the extracellular space due to the breakdown of hemoglobin. Following bleeding, a significant amount of iron is released from hemoglobin into the interstitial space, resulting in the generation of free radicals through the Fenton reaction and, as a result, oxidative destruction to protein, lipids, and DNA (Figures 2 and 3). The concentrations of NTBI in the cerebrospinal fluid of preterm with posthemorrhagic ventricular dilatation were established [88]. Administering erythrocyte suspension and its breakdown products causes ventricular dilatation, brain injury, and increased HO-1 enzyme and ferritin levels in rats, contributing to an increase in iron overload in the brain [89, 90].

## 3. Conclusions and future perspectives

For a child to develop to its full potential, programmed cell death must occur and it occurs more frequently in newborns than in adults [30, 39, 48]. Moreover, the cellular iron disparity is noticeable in neonates. Their antioxidant capacity is insufficient, implying that ferroptosis might also be significant in infants under pathologic conditions, despite the relatively small number of studies examining ferroptosis in neonatal brain injury [18–20, 58].

Neonatal brain injury treatment approaches for the term and preterm newborns are currently being developed; therefore, a thorough understanding of their mechanisms is critical to their success. Ferroptosis in neonatal brain injury models should be investigated further. In contrast to non-CNS tissues, iron chelators must transmit the BBB to reach the CNS parenchyma. Then, they must invade iron-accumulating cells and retrieve iron from the labile iron pool and ferritin, and the iron–chelator complex must depart the cells via the vasculature. Iron chelators have been reported to be effective in certain neurological conditions. The mechanism by which these chelators eliminate iron from the CNS remains to be elucidated and requires additional research [18,19,42,48].

Even with the therapeutic aim, many clinical applications could be reviewed considering the potential microglial involvement, ferroptosis, and possible clinical and neurodevelopmental outcomes. Common utilization of iNO in both term and preterm infants may also be considered an unknown characterized sword. Comprehensive and precise studies of the molecular dimensions of cellular iron storage and transfer could significantly contribute to identification of alternative strategies for preventing and treating brain injury in neonates
